# Brain and Cardiometabolic Effects of Major Weight Loss in People With Obesity: Preliminary Cohort Findings

**DOI:** 10.1002/osp4.70191

**Published:** 2026-10-30

**Authors:** Naomi L. Gaggi, Brian Hurwitz, Jennifer Bernal, Courtney O'Brien, Christine Ren‐Fielding, Holly Lofton, Jenny Chen, Aviezer Saperstein, Sang Han Lee, Yun‐Juan Chang, Kathy F. Yates, Els Fieremans, Ricardo Osorio, Antonio Convit

**Affiliations:** ^1^ Department of Psychiatry New York University Grossman School of Medicine New York New York USA; ^2^ Department of Clinical Research Nathan S. Kline Institute for Psychiatric Research New York New York USA; ^3^ Department of Bariatric Surgery New York University Langone Health New York New York USA; ^4^ Department of Radiology New York University Grossman School of Medicine New York New York USA; ^5^ Nathan S. Kline Institute for Psychiatric Research Center for Dementia Research New York New York USA

**Keywords:** Alzheimer's disease, cardiometabolic, neuroimaging, obesity, weight loss

## Abstract

**Objective:**

Given the overlap between obesity‐related metabolic and vascular abnormalities and Alzheimer's disease (AD) risk factors, it is critical to determine whether weight loss improves brain and cardiovascular health. This study examined whether ≥ 40% excess‐weight loss is associated with improvements in cardiometabolic risk, brain structure, and cognition.

**Methods:**

The study was a longitudinal study of individuals with obesity undergoing metabolic and bariatric surgery or medical/behavioral weight‐loss treatment. Participants were classified at follow‐up as weight‐loss (WL; ≥ 40% excess‐weight loss) or non‐weight‐loss (nWL; < 40%). All completed medical evaluations, structural MRI, and cognitive testing at baseline and 1 year.

**Results:**

At 1 year, the WL group exhibited improvements in BMI, HbA1C, inflammatory, metabolic, and lipid profiles. MRI‐based volumetrics revealed increases in subcortical gray‐matter volume and preservation of AD‐vulnerable regions. Cognitive performance remained unchanged.

**Conclusions:**

Achieving ≥ 40% excess‐weight loss was associated with improvements in metabolic health and brain structure, including favorable subcortical volume increases. These findings underscore the potential of sustained weight reduction to mitigate risks linked to metabolic syndromes and neurodegeneration. Longer‐term follow‐up is warranted to determine whether continued metabolic improvements lead to detectable cognitive gains and additional brain‐health benefits beyond 1 year.

## 1 Introduction

If current trends persist, by 2050 an additional ∼7 million individuals aged 5–24 years and ∼41 million adults (≥ 25 years) are projected to have obesity [1]. Obesity is a major driver of cardiometabolic disorders, including type 2 diabetes (T2D), hypertension, dyslipidemia, endothelial dysfunction, and chronic inflammation, which promote cerebrovascular injury and brain atrophy [2, 3, 4, 5, 6, 7, 8, 9, 10, 11, 12]. Although many associations among obesity, metabolic dysregulation, and brain abnormalities appear to be mediated by increased cerebrovascular disease [10, 13, 14, 15, 16], similar patterns are observed in youth, long before overt vascular pathology, suggesting additional mechanisms are involved [17, 18]. Whether obesity‐associated brain abnormalities, once established, can be reversed through weight loss remains unknown. This question is increasingly relevant, as the use of weight‐loss medications in the United States doubled from 0.76 million to 1.51 million users between 2017 and 2024 [19], continuing to rise very rapidly, and these medications have been associated with a decrease in dementia cases [20]. This study aimed to determine whether weight loss, and its associated reductions in metabolic syndrome, inflammation and insulin resistance, are linked to improvements in brain structure.

Although as little as 5% weight loss improves blood pressure, blood sugar levels, and lipid profiles [21, 22], greater weight loss is associated with greater improvement in these risk factors for cardiovascular disease [23]. Cardiometabolic diseases are linked to Alzheimer's disease (AD) through shared risk factors, underlying biological mechanisms, and genetics. Given the overlap in vascular and metabolic risk factors contributing to both cardiometabolic disease and AD pathology, substantial weight reduction may provide a mechanistic pathway linking metabolic improvements to cognitive outcomes [24]. As strategies for significant weight loss continue to become available [25, 26, 27], there is a major need to understand how weight loss and improvements in the associated cardiometabolic risk factors may lead to brain improvements and future cognitive health, particularly among those with T2D or significant insulin resistance.

This prospective, longitudinal cohort study tested the effect of large weight loss on the central nervous system and systemic outcomes. By evaluating a cohort of individuals with obesity seeking weight loss treatment, the study compared patients with and without 40% or greater excess weight loss, regardless of whether the weight loss was due to surgery or medical/behavioral treatment. This design enabled adjustment for baseline clinical, demographic, and metabolic covariates, strengthening causal inference. Participants underwent comprehensive assessments of brain structure, function, and metabolic health at baseline and one‐year follow‐up. By integrating these data, the study aimed to determine whether major weight loss produces measurable improvements in brain health and identify mechanisms that may mediate those changes.

## 2 Methods

### 2.1 Participants

This study was conducted at the New York University Grossman School of Medicine's Brain, Obesity & Diabetes Laboratory (BODy Lab). This study was approved by the IRB at New York University Grossman School of Medicine (S17‐01236). The cohort of individuals with obesity was enrolled in the baseline evaluation from 2018 to 2023. In general, the follow‐up time for each participant was 1 year, at which time they received an evaluation identical to the baseline evaluation. 84 subjects completed the study and had baseline and follow‐up data. Thirty individuals were retrospectively assigned to the non‐weight loss group (nWL) and 54 assigned to the weight loss group (WL).

### 2.2 Eligibility and Group Assignment

Participants were recruited from two weight loss programs, NYU Langone Medical Center or NYU Langone Brooklyn. To be eligible to participate in this study, participants had to meet the following criteria: ages 33–59, a BMI between 30 and 50 kg/m^2^, and a diagnosis of T2D (fasting glucose level ≥ 126 mg/dL or HbA1c ≥ 6.5%), pre‐diabetes (HbA1C of 5.7%–6.4%, or fasting glucose level 100 mg/dL–125 mg/dL), or insulin resistance as ascertained by a Quantitative Insulin Sensitivity Check Index (QUICKI) of ≤ 0.35. Both weight loss programs provided medical/behavioral regimens, but the bulk of subjects elected to undergo metabolic and bariatric surgery.

Although it was initially expected that the surgical group would achieve greater weight loss, the rapid uptake of the new weight‐loss medications during the study introduced substantial variability across treatment types. Therefore, participants were categorized post hoc based on weight‐loss magnitude rather than intervention type. Excess weight was calculated as the difference between each participant's actual weight and the weight corresponding to a BMI of 25 kg/m^2^. Individuals with ≥ 40% excess weight loss were assigned to the weight‐loss group (WL) and those who lost < 40% excess weight to the non‐weight loss group (nWL; Table 1). Although these cutoffs were arbitrary, they were chosen to be conservative and to optimize baseline weight distribution and demographic characteristics. There were 45 subjects that did not have two evaluations and/or imaging data, who were excluded from this report. There were no differences in the descriptive characteristics between the subjects included in the report and those with incomplete data.

**TABLE 1:** Weight change characteristics for the WL and nWL groups.

Group	Weight baseline (lbs)	Weight follow‐up (lbs)	Excess weight baseline (lbs)	Excess weight follow‐up (lbs)	% excess weight loss (PEWL)	% total weight loss (PTWL)
nWL	244.57 ± 37.90	235.73 ± 35.03	84.67 ± 27.44	75.83 ± 26.47	9.71 ± 16.73	3.38 ± 5.50
WL	245.42 ± 39.21	186.51 ± 28.47	93.55 ± 30.72	34.64 ± 25.32	64.39 ± 18.63	23.62 ± 6.88

Treatment approaches included metabolic and bariatric surgery and behavioral/medical weight‐loss interventions across both the WL and nWL groups. In the overall cohort (*N* = 84), 57 participants underwent bariatric surgery, including sleeve gastrectomy (*n* = 46), Roux‐en‐Y gastric bypass (*n* = 5), and laparoscopic adjustable gastric banding (*n* = 6), while 27 participants received behavioral/medical weight‐loss interventions. Within the WL group, 50 participants underwent bariatric surgery (sleeve gastrectomy, *n* = 41; Roux‐en‐Y gastric bypass, *n* = 5; laparoscopic adjustable gastric banding, *n* = 4), and 4 received behavioral/medical interventions. Within the nWL group, 7 participants underwent bariatric surgery (sleeve gastrectomy, *n* = 5; gastric banding, *n* = 2), while 23 received behavioral/medical interventions. GLP‐1 receptor agonists were used as adjunctive weight‐loss therapy by 25 participants across both treatment approaches, including 9 participants in the surgical group and 16 in the behavioral/medical group.

### 2.3 Clinical Assessments

Participants underwent comprehensive medical and imaging evaluations consisting of a physical examination, blood collection for labs and biomarkers, MRI scan, and cognitive assessments. The physical exam, including vital signs, was conducted by research nurses. Measurements included height, weight, blood pressure, radial pulse, shoulder width, and waist circumference measured at the umbilicus.

Blood draws were performed with individual fasting overnight and blood was processed for standard labs, and inflammatory markers. Participants also underwent a 5‐sample oral glucose tolerance test (OGTT) to ascertain glucose control and estimate insulin sensitivity. Research nurses obtained a blood sample via an intravenous line at thirty‐minute intervals over a 120‐min period following ingestion of either 50 g or 70 g of Glucola, ensuring each subject received the same dose at both evaluations. Insulin Sensitive Index (ISI) was calculated by ISI = exp[3.29–0.25*ln(fasting insulin) −0.22*ln(BMI) −0.28*ln(triglycerides)]. Metabolic syndrome was defined using the harmonized Joint Interim Statement criteria as the presence of ≥ 3 of 5 components: elevated waist circumference, elevated triglycerides, reduced HDL cholesterol or LDL targeting medication, elevated blood pressure or antihypertensive treatment, and elevated fasting glucose or glycemic treatment [28]. Participants were also asked to complete a number of self‐administered questionnaires assessing physical activity, weight history, mood, subjective memory performance, and sleep habits.

### 2.4 Neurocognitive Assessment

Participants underwent a comprehensive, standardized neuropsychological assessment including memory, attention, executive function, and processing speed. Cognitive performance did not differ between groups at baseline or follow‐up. Because cognitive outcomes remain a focus of ongoing research in the weight‐loss field, full descriptions of cognitive measures, scoring procedures, and detailed results are provided to support future investigation and meta‐analytic efforts (see Supporting Information S1: Cognitive Testing; Supporting Information S1: Table S1 and S2).

### 2.5 MRI Acquisition

Each participant underwent two identical standardized research neuroimaging evaluations approximately 1 year apart. All magnetic resonance imaging (MRI) was conducted on Siemens 3T Magnetom Biograph. The neuroimaging session comprised of both FDG‐PET and MRI (structural, functional) scans. However, this report only focuses on the 3D structural T1 MP2RAGE scan. The structural MP2RAGE was used for tissue segmentation and T1 quantitative mapping with the following parameters: voxel size = 1.0 mm^3^ isotropic, TR = 3130 ms, TE = 2.98 ms, TI1 = 900 ms, TI2 = 2500 ms, Flip Angels 1 & 2 = 9^o^, FOV = 256 mm, GRAPPA = 2, total time = 7:17 minutes. For a full description of the neuroimaging protocol, please refer to the Supplemental Materials (Supplemental Materials: Full Magnetic Resonance Imaging Protocol).

### 2.6 MRI Analysis

Structural T1‐weighted images were processed with FreeSurfer version 7.1.1 using the longitudinal pipeline, which includes intensity normalization, bias field correction, skull stripping, Talairach alignment, subcortical segmentation (aseg), cortical surface reconstruction with automated topology correction, and cortical parcellations. Structural scans were visually inspected for quality, motion, and artifacts. Of the participants who completed baseline and follow‐up evaluations, two participants were excluded from structural analyses due to susceptibility‐related distortion artifacts.

In addition, quantitative T1 maps were generated from the MP2RAGE acquisition using a Python implementation based on the MP2RAGE model [29]. These T1 maps were generated due to the potential influence of obesity on T1 relaxometry and consequently on structural segmentation [30]. In short, the MP2RAGE method estimates voxel‐wise T_1_ by acquiring two GRE readouts at different inversion times and combining them into a bias‐reduced uniform (UNI) image using a ratio of the real part of their product to the sum of their squared magnitudes. The resulting UNI image was clipped to the interval −1 to 1 to ensure numerical stability. A Bloch‐equation forward model of the MP2RAGE sequence, parameterized by the acquisition settings (inversion times, flip angles, repetition times, and inversion efficiency), is then used to simulate UNI values across a physiologic T_1_ range and build a dense look‐up table. Voxel‐wise T_1_ values are obtained by interpolating the measured UNI against this table. This procedure yielded quantitative T1 maps in seconds alongside the UNI image, which were subsequently used in downstream analysis. T1 relaxation times were extracted in milliseconds within the total cortical thickness and total subcortical gray matter regions using the FreeSurfer outputs described above (for more information please see Supporting Information S1: T1 Relaxometry).

Structural segmentations included cortical (aparc), subcortical (aseg), intracranial vault volume, and total subcortical gray matter volume FreeSurfer outputs, which were used as volumetric inputs for further analyses. In addition, this study focused on AD‐vulnerable regions including the bilateral inferior parietal cortices, posterior cingulate cortex, inferior temporal cortices, and hippocampus [31]. These regions were examined as a mean mask of all regions (AD vulnerable regions) and separately as whole regional values. AD‐vulnerable regions are particularly sensitive to metabolic, vascular, and inflammatory insults that are also prevalent in obesity. Examining these regions in a population with obesity before and after weight loss provides an opportunity to test whether structural changes associated with obesity overlap with brain networks implicated in AD risk. By focusing on AD‐sensitive regions, these analyses can assess whether weight loss may confer neuroprotective effects in areas most susceptible to long‐term cognitive decline.

### 2.7 Demographic Measures

Demographic information was obtained during the participant screening call and the first baseline evaluation. Participants provided their age, sex, race, native language, educational history, employment status, and some information about the household in which they grew up in. To measure socioeconomic status, the Hollingshead Index survey [32] was utilized by weighting occupation and education scale scores with a score range of 8–66. Social status scores were calculated as (occupational score × 5) + (educational score × 3). Individuals were assigned to one of five social classes: upper (66–55), upper‐middle (54–40), middle (39–30), lower‐middle (29–20), and lower (19–8).

### 2.8 Statistical Analyses

At baseline, demographic, clinical, cognitive, and brain structure (residualized to intracranial vault volume (eTIV)) characteristics were compared between the WL and nWL groups using t‐tests (normal distributions with unequal variances). For additional longitudinal analyses of changes between the two time points by groups, linear mixed effects models (using lmer function in R) were conducted. For clinical, cognitive, and demographic measures, covariates included age at baseline and time point and group as factors.

For brain structure measures, all values were first residualized to eTIV and covariates included age at the time of scan and the interval (in days) between baseline and follow‐up scans (Supporting Information S1: Table S3) with group as the factor. This was conducted twice, with and without T1 relaxation time (ms) as a covariate, given the potential influence of T1 relaxometry on obesity and structural segmentation [30].

To examine within group changes over time, paired t‐tests were conducted in the nWL and WL groups for all variables. Partial correlations were then conducted with change scores of all clinical, cognitive, and brain variables, percent excess weight loss (PEWL), and percent total weight loss (PTWL) across both the WL and nWL groups, while controlling for the covariates described above (Supporting Information S1: Table S4). The PEWL and PTWL were hypothesized to be better indicators of change in weight than a simple subtraction of weight at each time point as these accounts for baseline weight. Metabolic syndrome and sex were compared between the groups using Fischer's Exact Test. All statistical analyses were conducted in R (v.4.5.1) [33]. Uncorrected *p*‐values are reported throughout and if any tests survived FDR correction, this was noted.

## 3 Results

### 3.1 Baseline Analyses

#### 3.1.1 Demographic and Clinical Characteristics

The study included 84 participants split into the WL (*n* = 54; mean baseline BMI = 38.24; APOE4 28%; 50% Hispanic; 24.07% Black or African American; 18.52% White; 7.41% Other) and nWL (*n* = 30; mean baseline BMI = 40.40; APOE4 41%; 20% Hispanic; 56.67% Black or African American; 10% White; 13.33% Other) groups (see Table 2). There was no significant difference in sex distribution between groups at baseline (OR = 0.48, 95% CI [0.14, 1.69], *p* = 0.247). The nWL group consisted of 73.3% females, while the WL group consisted of 85.2% females. Within the WL group, 3.77% had non‐surgical weight loss and 96.23% underwent surgery. Within the nWL group, 68.97% had non‐surgical weight loss and 31.03% underwent surgery. The groups were matched for age (nWL: 45.73 ± 6.07, WL: 44.59 ± 7.11; *t* = 0.078, *p* < 0.441), education nWL: 15.73 ± 1.78 years, WL: 15.61 ± 1.91; *t* = 0.303, *p* < 0.673), Hollings Head Index (nWL: 49.83 ± 9.51, WL: 50.33 ± 10.32; *t* = −0.22, *p* < 0.924), weight (*t* = −0.10, *p* < 0.923), excess weight (*t* = −1.36, *p* < 0.178), Waist (nWL: 119.48 ± 11.43, WL: 118.59 ± 14.83, *t* = 0.30, *p* = 0.76), Waist to Height Ratio (nWL: 0.73 ± 0.07, WL: 0.71 ± 0.10, *t*(73.44) = 1.06, *p* = 0.292), and metabolic syndrome (nWL: 83.3%, WL: 68.5%; OR = 0.439, 95% CI [0.11–1.45], *p* = 0.196; Supporting Information S1: Table S6).

**TABLE 2:** Demographics and clinical variables at baseline and 1 Year between groups.

Variable	nWL baseline (Mean ± SD)	nWL follow up (Mean ± SD)	WL baseline (Mean ± SD)	WL follow up (Mean ± SD)	Between group		Group × time interaction					
					*t*	*p*	*B*_3_	*F*	df1	df2	*p*	η^2^_p_
BMI	38.25 ± 4.13	37.19 ± 3.93	40.40 ± 4.65	30.89 ± 3.71	−2.19	0.032*	−8.45	124.65	1	82	< 0.001****	0.603
CRP	7.65 ± 9.96	6.98 ± 8.90	10.09 ± 7.76	2.89 ± 2.92	−1.12	0.265	−6.53	25.14	1	82	< 0.001****	0.235
A1C	6.07 ± 0.72	5.93 ± 0.92	5.97 ± 0.52	5.28 ± 0.27	0.64	0.528	−0.55	13.83	1	82	< 0.001****	0.144
Systolic BP	126.05 ± 12.3	122.19 ± 11.9	124.19 ± 15.9	119.84 ± 16.4	0.84	0.406	−0.49	0.02	1	82	0.896	0.000
Diastolic BP	77.54 ± 9.56	75.38 ± 9.90	75.68 ± 11.4	73.31 ± 11.5	0.88	0.382	−0.20	0.01	1	82	0.939	0.000
Glucose fasting	103.70 ± 19.0	106.33 ± 24.5	106.06 ± 28.3	87.48 ± 9.59	−0.47	0.636	−21.21	12.81	1	82	< 0.001****	0.135
Insulin fasting	14.81 ± 9.58	13.23 ± 8.01	17.28 ± 9.75	7.37 ± 3.06	−1.11	0.270	−8.32	18.33	1	82	< 0.001****	0.183
QUICKI	0.329 ± 0.043	0.332 ± 0.040	0.319 ± 0.037	0.363 ± 0.025	1.08	0.284	0.040	18.39	1	82	< 0.001****	0.183
HOMA‐IR	4.00 ± 3.09	3.57 ± 2.55	4.65 ± 2.98	1.61 ± 0.77	−0.94	0.353	−2.61	17.04	1	82	< 0.001****	0.172
ISI	1.87 ± 0.53	1.92 ± 0.47	1.70 ± 0.47	2.29 ± 0.48	1.44	0.157	0.54	21.41	1	82	< 0.001****	0.207
Triglycerides	112.35 ± 74.2	106.28 ± 55.2	122.18 ± 68.1	98.58 ± 61.5	−0.55	0.582	−17.53	2.69	1	82	0.105	0.032
HDL cholesterol	43.17 ± 10.6	47.60 ± 12.2	44.33 ± 6.81	54.96 ± 11.8	−0.39	0.691	6.20	7.73	1	82	0.007**	0.086
LDL cholesterol	100.42 ± 27.1	111.36 ± 33.7	120.19 ± 33.5	115.43 ± 28.8	−2.82	0.006**	−15.70	8.61	1	81	0.004***	0.096
Chol/HDL ratio	3.97 ± 1.05	3.93 ± 0.95	4.31 ± 1.02	3.63 ± 1.03	−2.25	0.027*	−0.64	12.84	1	82	< 0.001****	0.135
Waist (cm)	119.48 ± 11.43	116.15 ± 11.51	118.59 ± 14.83	96.42 ± 13.75	0.30	0.761	−18.84	52.73	1	82	< 0.001****	0.391
Waist‐to‐height ratio	0.73 ± 0.07	0.71 ± 0.07	0.71 ± 0.10	0.57 ± 0.09	1.06	0.292	−0.11	52.92	1	82	< 0.001****	0.392
IL‐10	2.64 ± 1.26	3.42 ± 5.83	2.52 ± 1.07	2.46 ± 1.54	0.38	0.704	−0.85	0.95	1	79	0.332	0.012
IL‐6	4.90 ± 2.45	4.03 ± 2.13	5.94 ± 3.30	4.74 ± 8.81	−1.58	0.119	−0.34	0.04	1	82	0.834	0.001
Adiponectin	7.53 ± 5.47	8.12 ± 6.00	9.86 ± 5.93	14.27 ± 10.74	−1.67	0.101	3.83	5.57	1	82	0.018*	< 0.000
AST	22.87 ± 7.60	23.40 ± 11.60	25.65 ± 11.82	20.24 ± 6.91	−1.31	0.194	−5.94	4.59	1	82	0.035*	0.053
ALT	22.83 ± 10.14	21.80 ± 9.93	33.13 ± 24.44	18.13 ± 9.26	−2.70	0.008**	−14.02	8.29	1	82	0.005****	0.093
Thyroid stimulating hormone (TSH)	1.38 ± 0.79	1.60 ± 0.90	1.70 ± 1.08	1.39 ± 0.90	−1.56	0.124	7.25	5.06	1	81	0.009***	0.083

^*^ indicates *p* < 0.05.

^**^ indicates *p* < 0.01.

^***^ indicates *p* < 0.005.

^****^ indicates *p* < 0.001.

At baseline, few characteristics distinguished the groups by t‐tests, with the exception of higher BMI (*t* = −2.19, *p* = 0.032), LDL cholesterol (*t* = −2.82, *p* = 0.006), cholesterol ratio (*t* = −2.25, *p* = 0.027), and alanine aminotransferase (ALT; *t* = −2.70, *p* = 0.008). All other variables, including insulin sensitivity indices, inflammatory cytokines, and sociodemographic factors, did not differ significantly between the groups. At baseline, the distribution of prescribed medications differed modestly between the groups. In the nWL group, 46.7% were on antihypertensives, 66.7% on glycemic, and 23.3% on lipid‐lowering therapies, compared with 38.9%, 33.3%, and 18.5%, respectively, in the WL group. Baseline inflammatory markers, including IL‐10, IL‐6, TNF‐α, CRP, and adiponectin, did not differ significantly between groups (*p* > 0.05). After controlling for multiple comparisons, the WL and nWL groups differed at baseline only in LDL cholesterol and ALT, while all other baseline clinical variables were statistically comparable between the groups. These results indicate that the WL and nWL groups were well‐matched.

#### 3.1.2 Brain Structure

Regional cortical and subcortical volumes were compared between the WL and nWL groups at baseline to assess comparability prior to weight‐loss–related changes. Volumetric measures were residualized to estimated intracranial volume (eTIV), and t‐tests were performed across seven regions of interest. Small and trending group differences were observed in the inferior parietal cortex (*t* = 2,40, *p* = 0.019), inferior temporal cortex (*t* = 2.56, *p* = 0.013), and an AD‐vulnerable cortical composite (*t* = 2.78, *p* = 0.007; Table 3). No significant differences were detected in the lateral occipital cortex, hippocampus, or total cortical gray matter. These results all survived the FDR correction. Overall, these results indicate that the groups were broadly comparable in regional brain structure at study entry.

**TABLE 3:** Regional brain volumetric measures (mm^3^) at baseline and 1 year between groups.

Variable	nWL baseline (Mean ± SD)	nWL follow up (Mean ± SD)	WL baseline (Mean ± SD)	WL follow up (Mean ± SD)	Between group		Group × time interaction					
					*t*	*p*	*B*_3_	*F*	df1	df2	*p*	η^2^_p_
Cortical gray matter	465,234 ± 41,534	462,365 ± 38,959	448,032 ± 37,271	446,423 ± 36,231	1.82	0.075	521.39	0.05	1	80	0.822	0.001
Inferior parietal	26,832 ± 2912	26,640 ± 2702	25,125 ± 3300	25,082 ± 3139	2.40	0.019*	54.21	0.60	1	80	0.440	0.008
Inferior temporal	21,548 ± 2635	21,314 ± 2567	19,987 ± 2598	19,966 ± 2645	2.56	0.013*	120.28	1.70	1	80	0.197	0.021
Lateral occipital	22,820 ± 2938	22,699 ± 2937	22,825 ± 3059	23,272 ± 2894	−0.01	0.994	266.74	3.35	1	80	0.071	0.041
Posterior cingulate	6065 ± 827	6021 ± 838	5838 ± 691	5834 ± 654	1.25	0.216	17.19	0.82	1	80	0.368	0.010
Total hippocampus	8382 ± 913	8398 ± 881	8510 ± 805	8494 ± 824	−0.63	0.532	−9.98	0.18	1	80	0.676	0.002
AD‐vulnerable composite	54,723 ± 5674	54,163 ± 5434	50,950 ± 5766	50,881 ± 5600	2.78	0.007**	472.22	2.48	1	80	0.119	0.032
Subcortical gray matter	60,003 ± 4956	60,101 ± 4751	59,083 ± 4802	59,811 ± 4776	0.81	0.423	618.96	6.01	1	80	0.016*	0.072

^*^ indicates *p* < 0.05.

^**^ indicates *p* < 0.01.

*** indicates *p* < 0.005.

**** indicates *p* < 0.001.

## 4 Longitudinal Analyses

### 4.1 Metabolic and Inflammatory Changes

Mixed effects model results (Table 2) revealed, as expected, that WL participants exhibited significantly greater reductions from baseline to follow‐up in BMI (*F*(1, 82) = 124.65, *p* < 0.001, η^2^_p_ = 0.603), waist circumference (cm); (F(1, 82) = 52.73, *p* < 0.001, η^2^_p_ = 0.391), waist to height ratio (F(1, 82) = 52.92, *p* < 0.001, η^2^_p_ = 0.392), as well as improved A1C (*F*(1, 82) = 13.83, *p* < 0.001, η^2^_p_ = 0.144). Significant interactions also emerged for decreased fasting glucose, fasting insulin, HOMA‐IR, CRP (all *p* < 0.001), TSH, and adiponectin (both *p* < 0.05) in the WL group and increased ISI and QUICKI (both *p* < 0.001), indicating greater metabolic improvement in the WL group relative to nWL group. In lipid markers, significant interactions were detected for improved HDL cholesterol (increased), LDL cholesterol (decreased), and the HDL/cholesterol ratio (decreased; all *p* < 0.007), reflecting a healthier lipid profile over time among WL participants. For liver enzymes, significant group × time effects were observed for decreased ALT and aspartate aminotransferase, suggesting improved hepatic function following weight loss. No significant interactions were found for systolic or diastolic blood pressure (both *p* > 0.05).

After FDR correction, the Group × Time interaction remained significant for BMI, CRP, A1C, fasting glucose, fasting insulin, QUICKI, HOMA‐IR, ISI, cholesterol/HDL ratio, LDL cholesterol, HDL cholesterol, ALT, AST, adiponectin, and TSH. In addition to group differences, the PEWL and PTWL changes across all subjects were strongly associated with improvements in multiple metabolic markers (Supporting Information S1: Tables S4 and S5). Within‐group changes revealed improvements in the WL group, whereas the nWL group demonstrated increased HDL and LDL cholesterol in addition to IL‐6 (Table 4). Only changes within the WL group survived multiple comparison corrections, not the nWL group. In addition, at follow‐up, the prevalence of metabolic syndrome was significantly lower in the WL group than in the nWL group (OR = 0.17, 95% CI [0.06–0.49], *p* = 0.0003; Supporting Information S1: Table S6).

**TABLE 4:** Demographic and clinical variable within‐group changes.

Variable	Group	*t*	*p*
BMI	nWL	2.64	0.013*
BMI	WL	18.3	< 0.001****
CRP	nWL	0.865	0.394
CRP	WL	8.29	< 0.001****
A1C	nWL	0.895	0.378
A1C	WL	9.60	< 0.001****
Systolic BP	nWL	1.28	0.210
Systolic BP	WL	1.99	0.052
Diastolic BP	nWL	1.01	0.319
Diastolic BP	WL	1.50	0.139
Glucose fasting	nWL	−0.518	0.608
Glucose fasting	WL	5.47	< 0.001****
Insulin fasting	nWL	1.28	0.212
Insulin fasting	WL	7.79	< 0.001****
QUICKI	nWL	−0.388	0.701
QUICKI	WL	−8.57	< 0.001****
HOMA‐IR	nWL	0.903	0.374
HOMA‐IR	WL	7.79	< 0.001***
Weight (lbs)	nWL	3.44	0.002***
Weight (lbs)	WL	19.3	< 0.001****
Excess weight baseline (lbs)	nWL	3.44	0.002***
Excess weight baseline (lbs)	WL	19.3	< 0.001****
Triglycerides	nWL	0.525	0.604
Triglycerides	WL	4.98	< 0.001****
HDL cholesterol	nWL	−3.00	0.005***
HDL cholesterol	WL	−7.37	< 0.001****
LDL cholesterol	nWL	−2.52	0.017**
LDL cholesterol	WL	1.57	0.123
Cholesterol ratio	nWL	0.321	0.751
Cholesterol ratio	WL	6.22	< 0.001****
IL‐10	nWL	−0.736	0.467
IL‐10	WL	−0.034	0.973
IL‐6	nWL	3.93	< 0.001****
IL‐6	WL	0.966	0.339
Adiponectin	nWL	−1.20	0.238
Adiponectin	WL	−3.64	< 0.001****
AST	nWL	−0.287	0.776
AST	WL	3.02	0.004***
ALT	nWL	0.698	0.491
ALT	WL	4.32	< 0.001****
TSH	nWL	1.40	0.171
TSH	WL	−2.54	0.014**
ISI	nWL	−0.457	0.651
ISI	WL	−9.08	< 0.001****

^*^ indicates *p* < 0.05.

^**^ indicates *p* < 0.01.

^***^ indicates *p* < 0.005.

^****^ indicates *p* < 0.001.

## 5 Brain‐Related Change

Subcortical gray volume (residualized to eTIV) showed significant group × time interactions after adjusting for age and days between scans (*p* = 0.016; Table 3). Interactions in the posterior cingulate, inferior parietal, inferior temporal, hippocampus, total cortical gray, and AD‐vulnerable composite were not significant (all *p* > 0.10). The lateral occipital region was trending toward significant (*p* < 0.071). Including cortical T1 relaxation time as an additional covariate did not alter these findings. These results suggest that total cortical gray matter volume change was not different between groups and was not influenced by cortical T1 relaxation changes.

The significant group × time interaction for subcortical gray matter volume remained significant when the change in T1 relaxation time between baseline and follow‐up was added to the model, with the WL group showing significantly greater increases in volume than the nWL group. Main effects of time, age, and eTIV were also significant (*p* < 0.01), while change in T1 subcortical relaxation time was not a significant covariate (*p* = 0.28). The T1 relaxation covariate itself was not significant, indicating that the observed group difference in subcortical volume change was robust to the inclusion of T1 variability. However, these changes do not survive the FDR correction. Within‐group comparisons, revealed significant increases in the subcortical gray matter in WL with no change to AD‐vulnerable regions, whereas there were significant decreases in AD‐vulnerable regions in the nWL group (Table 5).

**TABLE 5:** Regional brain volumetric measures (mm^3^) within‐group changes.

Variable	Group	*t*	*p*
AD vulnerable composite	nWL	2.66	0.013**
AD vulnerable composite	WL	0.364	0.717
Subcortical gray matter	nWL	−0.605	0.550
Subcortical gray matter	WL	−4.38	< 0.001****
Cortical gray matter	nWL	1.89	0.070
Cortical gray matter	WL	1.12	0.270
Inferior parietal cortex	nWL	2.00	0.055
Inferior parietal cortex	WL	0.472	0.639
Inferior temporal cortex	nWL	1.68	0.103
Inferior temporal cortex	WL	0.179	0.859
Lateral occipital cortex	nWL	1.16	0.256
Lateral occipital cortex	WL	−2.12	0.039**
Posterior cingulate cortex	nWL	1.62	0.117
Posterior cingulate cortex	WL	0.172	0.864
Hippocampus	nWL	−0.477	0.637
Hippocampus	WL	0.518	0.607

*indicates *p* < 0.05.

^**^ indicates *p* < 0.01.

*** indicates *p* < 0.005.

^****^ indicates *p* < 0.001.

## 6 Discussion

This study shows that the metabolic effects of significant weight loss are accompanied by improvements in physiologically relevant neuroimaging biomarkers. Specifically, the results showed that more excess weight loss was associated with: (1) increased volume in subcortical gray matter and (2) improvement in T1‐relaxometry of both cortical and subcortical regions. Alongside these neuroimaging effects, participants who lost more weight also demonstrated substantial improvements in glycemic control, lipid profiles, and inflammatory markers. This study is timely as the use of weight loss medication is rapidly increasing [19], highlighting the need to better characterize how weight loss impacts cardiometabolic risk factors relevant to AD.

At baseline, as expected, the WL and nWL groups were similar across most demographic, clinical, and metabolic variables. Notably, cognitive performance did not differ significantly between groups at baseline (see Supporting Information S1: Tables S1 and S2). Although we did not observe significant cognitive change over the follow‐up interval, prior longitudinal studies have reported improvements in neuropsychological performance after bariatric surgery, including gains in attention, executive function, and memory over follow‐up periods extending to 3 years [34, 35]. The absence of detectable cognitive improvement in the present cohort may reflect the shorter follow‐up interval, heterogeneity in weight‐loss response and baseline metabolic status, and/or limited sensitivity to detect cognitive change over approximately 1 year. Future longer‐term studies will be needed to determine whether structural brain changes after weight loss precede measurable cognitive benefits and whether changes in brain structure correlate with subsequent cognitive trajectories.

Consistent with existing literature [36], the WL group demonstrated substantial improvements across multiple metabolic and inflammatory markers. Analyses revealed that, compared to nWL, the WL group showed statistically significant reductions in BMI, HbA1C, LDL cholesterol, cholesterol:HDL ratio, and HOMA‐IR, alongside increases in HDL cholesterol and QUICKI even a year apart. Blood pressure did not change significantly between groups, which is likely related to this cohort being broadly treated with antihypertensives at baseline. Notably, CRP, a marker of systemic inflammation commonly elevated in individuals with obesity [37], was significantly more reduced in the WL group, suggesting meaningful reductions in inflammatory burden following weight reduction.

At baseline, significant group differences emerged in the AD‐vulnerable region composite, with the nWL group showing a higher overall volume than the WL group, perhaps given that the WL group had a non‐significant, slightly higher BMI at baseline. This finding is likely driven by group differences in the inferior temporal and parietal regions. This is consistent with previous cross‐sectional findings of individuals with obesity demonstrating smaller gray matter cortical volume compared with those without obesity [38]. While individual regional differences (e.g., cortical gray matter) trended toward significance, most did not survive multiple comparison corrections.

When within‐group longitudinal changes in AD‐vulnerable regions were investigated using paired t‐tests, there was a significant decline in AD‐vulnerable volume in the nWL group, whereas the WL group showed no change in AD‐vulnerable volume (Table 5). However, when testing between‐group comparisons controlling for age and scan interval, and adjusting for multiple comparisons, AD‐vulnerable region volume did not differ between groups. Given that between‐group effects were not significant after adjustment, these within‐group differences should be viewed as preliminary. In addition, these analyses were conducted with regional volumes (within each hemisphere) to potentially detect more nuanced changes that may be overlooked, but this also yielded very similar non‐significant results. However, the relative stability of AD‐vulnerable regions in WL compared to the decline in the nWL group raises the possibility of early structural preservation that merits investigation over longer follow‐up intervals and in studies with larger sample sizes.

One notable longitudinal group effect was that subcortical gray matter volume was significantly increased in the WL group relative to nWL, an effect that persisted after accounting for T1 relaxation time. Importantly, higher PEWL was associated with greater reductions (improvement) in cortical and subcortical T1 relaxation values, aligning with previous literature in adolescents with obesity [38]. T1 relaxometry can serve as an indicator of brain health with longer relaxation times (higher T1 values) indicating some kind of damage [30, 39, 40, 41]. Taken together, these results indicate that weight loss is associated with improvements in volume and possibly also the health of gray matter, particularly in subcortical regions. These findings are consistent with the literature that higher BMI is associated with gray matter atrophy [34, 35].

In contrast to metabolic and neuroimaging differences, cognition did not differ significantly between groups either at baseline or longitudinally. While the WL group showed numerically greater improvements in memory composite scores, these improvements did not reach statistical significance and were highly variable (Supporting Information S1: Table S2). One possibility is that the one‐year follow‐up period was insufficient for subtle brain changes to translate into measurable cognitive effects. In addition, given the limited sensitivity of cognitive measures to short‐term change and the modest sample size, detecting weight‐loss‐related cognitive effects likely requires larger, adequately powered cohorts, with longer follow‐up.

Taken together, these findings suggest that significant weight loss is accompanied by robust metabolic and inflammatory improvements, as well as early signals of structural brain preservation, particularly in AD‐vulnerable and subcortical regions. Parallel improvements in peripheral and central biomarkers raise the possibility that weight loss may influence neurobiological pathways relevant to neurodegenerative risk, even before measurable cognitive changes emerge. Because structural brain changes likely precede detectable cognitive effects, these results highlight the importance of longitudinal designs capable of tracking multi‐year trajectories. This integrative pattern of metabolic and neuroimaging benefits provides a strong rationale for extended follow‐up to determine the durability and functional relevance of these changes.

## 7 Limitations

This study has several limitations. First, it was non‐randomized, and group assignment was determined post hoc based on weight loss. While this approach allowed for the comparison of two groups that differed substantially in weight loss and were otherwise relatively balanced at baseline, the possibility of unmeasured confounding cannot be fully excluded. Second, the sample size limits the ability to conduct subgroup analyses. For example, the relatively low proportion of men prevented examination of potential sex‐dependent effects of weight loss. Third, because approximately three‐quarters of individuals seeking treatment for obesity in our clinics were women, the study cohort reflects this clinical population. Fourth, the one‐year follow‐up interval was designed to evaluate short‐term metabolic and brain structural changes. Longer follow‐up will be needed to determine whether weight loss alters the trajectory of cognitive decline and neurodegenerative risk.

## 8 Conclusions

This study demonstrates that significant weight loss not only confers significant metabolic and inflammatory benefits but also measurable structural brain gains, most notably in subcortical gray matter volume. However, no significant changes in cognition were detected within the one‐year follow‐up, which may suggest the need for longer follow‐up and larger sample sizes given the higher variability in measures of cognitive performance. Future work should consider longer follow‐up durations to further elucidate the relationship between weight loss and neurocognitive health.

## Author Contributions

N.L.G., B.H., J.B., C.O., R.O. and A.C. have contributed to the original writing of the manuscript. All authors have contributed to the editing. J.B., C.O., C.R.F., H.L., J.C., A.S., K.F.Y., E.F., R.O. and A.C. have contributed to data acquisition and/or study design. N.G., B.H., J.B., Y.C. and C.O. were involved in the analysis of data, and S.H.L. advised on statistical methods. All authors have contributed substantially, including contributing to the interpretation of the results, and have agreed to the final submitted version.

## Funding

This study was funded, in part, by the American Academy of Sleep Medicine Foundation Focused Projects for Junior Investigators Grant (328‐FP‐24 PI: Gaggi).

## Ethics Statement

This study was approved by the Institutional Review Board (IRB) at New York University Grossman School of Medicine (S17‐01236). All participants provided written informed consent prior to study participation. All study procedures were conducted in accordance with the ethical standards of the institutional review board and applicable ethical guidelines.

## Conflicts of Interest

The authors declare no conflicts of interest.

## Supporting information


Supporting Information S1

## Data Availability

The data that support the findings of this study are available from the corresponding author upon reasonable request.
