# Supplementary material for: Brain and Cardiometabolic Effects of Major Weight Loss in People With Obesity: Preliminary Cohort Findings
Source: Obes Sci Pract. 2026 Oct 30;12(6):e70191. doi: 10.1002/osp4.70191 (PMC13720752; doi:10.1002/osp4.70191)
Supplement: Supplementary file 1 [file OSP4-12-e70191-s001.docx]

Supplemental Materials.

**Cognitive Testing.** Cognitive testers were trained and supervised by a senior neuropsychologist, and the assessment was administered in a controlled environment. The cognitive domains of memory and learning were assessed using the Digit Symbol Substitution Test (DSST; Wechsler, 1997), California Verbal Learning Test (CVLT-II; Delis, Kramer, Kaplan & Ober 2000), and Logical Memory Test (Wechsler, 1987). Attention and psychomotor efficiency were evaluated using Digit Symbol Substitution Test, Digit Vigilance Test (Lewis & Rennick, 1979), Trail Making Test (Trail A; US War Department, 1944), and Number Span Forward and Backward (Wechsler, 1997). Executive functioning was measured using the Stroop (Stroop, 1935), Trail Making Test (Trail B; US War Department, 1944), and Controlled Oral Word Association Test (COWAT; Benton, Hamsher & Sivan, 1983). Throughout all the assessments, there were embedded measures assessing the participants’ effort to ensure the validity of the information collected. A composite score was obtained by summing the z-scores of tasks that belong in the cognitive domains of executive function, learning, memory, logical memory, attention, and psychomotor efficiency.

**Full Magnetic Resonance Imaging Protocol.** Data were acquired on a **Siemens MAGNETOM Biograph mMR** hybrid PET/MR scanner equipped with a 3 T magnet and a fully integrated PET detector ring. All scans used a 32-channel head coil with automatic coil selection and adaptive combine reconstruction. Participants underwent simultaneous PET and MRI acquisition following intravenous administration of [F-18]-fluorodeoxyglucose ([F-18 FDG]) to evaluate cerebral glucose metabolism in conjunction with high-resolution structural, diffusion, perfusion, and functional MRI. Image localization was first achieved using multi-plane scout sequences to ensure precise isocentric positioning. High-resolution structural data were obtained using a T1-weighted MP2RAGE sequence (TR = 3130 ms, TE = 2.98 ms, TI₁ = 900 ms, TI₂ = 2500 ms, flip angles = 9°/9°) with 1.0 mm³ isotropic resolution and GRAPPA = 2, producing bias-field-corrected T1 maps used for cortical morphometry and coregistration with PET data. T2-weighted FLAIR images (TR = 9000 ms, TE = 90 ms, TI = 2500 ms, flip = 150°) were acquired at 0.7 × 0.7 × 5.0 mm³ resolution (GRAPPA = 2) to enhance gray-white matter contrast and detect lesions or white-matter hyperintensities. Diffusion kurtosis imaging (DKI) was performed with a spin-echo EPI sequence (TR = 7900 ms, TE = 96 ms, voxel = 2.5 × 2.5 × 3.0 mm³) using 44–48 diffusion directions at b = 0 and 2500 s/mm² (GRAPPA = 2). Perfusion imaging was performed using pseudocontinuous arterial spin labeling (pCASL; TR = 6810 ms, TE = 23.68 ms, voxel = 1.7 × 1.7 × 4.0 mm³), 1500 ms bolus, and 3000 ms inversion time to derive quantitative cerebral blood flow maps. Resting-state BOLD fMRI (TR = 2400 ms, TE = 30 ms, flip = 80°) was acquired with 3.2 mm³ isotropic voxels over 180 volumes (~7 min) while subjects rested with eyes closed, providing functional connectivity measures complementary to metabolic PET data. PET emission data were acquired concurrently using a dynamic frame protocol initiated at tracer injection.

**T1 Relaxometry.** T1 relaxation served as a covariate, which did not show significance, indicating that the observed group difference in subcortical volume change is robust to the inclusion of T1 variability. For the cortical T1 relaxation model, the Group × Time interaction was significant (F(1, 72) = 4.32, p = 0.041, η²ₚ = 0.027), but was only trending in the subcortical T1 model (F(1, 72) = 3.75, p = 0.057, η²ₚ = 0.024). Also, higher PEWL scores were significantly associated with greater decreases in both cortical (mean change = −12.4 ± 80.4 ms; r = −0.29, p = .014) and subcortical (mean change = −10.9 ± 74.0 ms; r = −0.29, p = .014) T1 values from baseline to follow up. The WL group showed slightly higher cortical (571.4 ± 84.9 ms) and subcortical (538.6 ± 79.2 ms) T1 values than the nWL group (cortical: 544.9 ± 43.9 ms; subcortical: 514.1 ± 35.8 ms) at study entry. These differences provide additional context for baseline morphometric characterization but do not indicate major structural divergence between groups

Supplemental Table 1. Baseline cognition in weight loss and non-weight loss groups.

| Variable | t-statistic | p-value | Mean nWL | SD nWL | Mean WL | SD WL |
| --- | --- | --- | --- | --- | --- | --- |
| Executive Function Composite | 1.21 | 0.229 | 0.59 | 3.52 | -0.43 | 3.89 |
| Learning Composite | -0.44 | 0.659 | -0.09 | 1.36 | 0.07 | 1.83 |
| Memory Composite | 0.66 | 0.512 | 0.21 | 2.33 | -0.16 | 2.73 |
| Attention Composite | 0.98 | 0.329 | 0.21 | 1.49 | -0.13 | 1.61 |
| Psychmotor Composite | 0.04 | 0.970 | 0.01 | 1.69 | 0.0 | 1.08 |

Supplemental Table 2. Longitudinal cognitive changes in weight loss and non-weight loss groups.

| Variable | Effect | df | F | p | η²ₚ |
| --- | --- | --- | --- | --- | --- |
| Executive Function Composite | timepoint | 81.0 | 1.16 | 0.284 | 0.0142 |
| Executive Function Composite | group | 80.0 | 0.877 | 0.352 | 0.0108 |
| Executive Function Composite | age | 80.0 | 0.792 | 0.376 | 0.00981 |
| Executive Function Composite | timepoint × group | 81.0 | 0.507 | 0.478 | 0.00622 |
| Learning Composite | timepoint | 81.0 | 0.0323 | 0.858 | 0.000398 |
| Learning Composite | group | 80.0 | 0.150 | 0.699 | 0.00187 |
| Learning Composite | age | 80.0 | 2.19 | 0.143 | 0.0267 |
| Learning Composite | timepoint × group | 81.0 | 0.0166 | 0.898 | 0.000205 |
| Memory Composite | timepoint | 81.0 | 28.4 | <.000 | 0.259 |
| Memory Composite | group | 80.0 | 0.738 | 0.393 | 0.00914 |
| Memory Composite | age | 80.0 | 0.0484 | 0.826 | 0.000605 |
| Memory Composite | timepoint × group | 81.0 | 0.205 | 0.652 | 0.00252 |
| Attention Composite | timepoint | 81.0 | 0.717 | 0.399 | 0.00878 |
| Attention Composite | group | 80.0 | 0.325 | 0.570 | 0.00405 |
| Attention Composite | age | 80.0 | 4.92 | 0.0294 | 0.0579 |
| Attention Composite | timepoint × group | 81.0 | 0.197 | 0.658 | 0.00243 |
| Psychmotor Composite | timepoint | 81.0 | 0.293 | 0.590 | 0.00361 |
| Psychmotor Composite | group | 80.0 | 0.171 | 0.680 | 0.00214 |
| Psychmotor Composite | age | 80.0 | 5.96 | 0.0169 | 0.0693 |
| Psychmotor Composite | timepoint × group | 81.0 | 0.112 | 0.738 | 0.00139 |

Supplemental Table 3. Mean Time Between Baseline and Follow Up Scans.

| Group | Days Between Scans (Mean ± SD) |
| --- | --- |
| nWL | 394.6 ± 184.8 |
| WL | 404.1 ± 124.2 |

Supplemental Table 4. Percent Excess Weight Loss Correlated with Clinical and Brain Changes

| Variable | Spearman rho | p | p_FDR |
| --- | --- | --- | --- |
| bmi_change | -0.843 | <0.001* | <0.001* |
| ast_change | -0.146 | 0.188 | 0.196 |
| alt_change | -0.370 | <0.001* | <0.001* |
| isi_bmi_change | 0.646 | <0.001* | <0.001* |
| Chol Ratio_change | -0.537 | <0.001* | <0.001* |
| CRP_change | -0.551 | <0.001* | <0.001* |
| A1C_change | -0.450 | <0.001* | <0.001* |
| systolic_change | -0.245 | 0.026* | 0.030* |
| diastolic_change | -0.146 | 0.189 | 0.196 |
| Glucose Fasting_change | -0.584 | <0.001V | <0.001* |
| Insulin Fasting_change | -0.520 | <0.001* | <0.001* |
| QUICKI_change | 0.631 | <0.001* | <0.001* |
| HOMA-IR_change | -0.533 | <0.001* | <0.001* |
| triglycerides_change | -0.388 | <0.001* | <0.001* |
| hdl_cholesterol_change | 0.331 | 0.002* | 0.0027* |
| ldl_cholesterol_change | -0.414 | <0.001* | <0.001* |
| cholesterol_hdl_ratio_change | -0.501 | <0.001* | <0.001* |
| IL-10_change | 0.159 | 0.160* | 0.179 |
| IL-6_change | -0.372 | <0.001* | <0.001* |
| adiponectin._change | 0.418 | <0.001* | <0.001* |
| Cortical Gray Matter | -0.045 | 0.693 | 0.905 |
| Inferior Parietal | -0.030 | 0.794 | 0.905 |
| Inferior Temporal | 0.002 | 0.984 | 0.984 |
| Lateral Occipital | 0.066 | 0.562 | 0.905 |
| Posterior Cingulate | 0.066 | 0.560 | 0.905 |
| Total Hippocampus | -0.184 | 0.102 | 0.408 |
| AD-Vulnerable Composite | 0.028 | 0.811 | 0.905 |
| Subcortical Gray Volume | 0.250 | 0.026* | 0.208 |

Supplemental Table 5. Percent Total Weight Loss Correlated with Clinical and Brain Changes

| Variable | Spearman rho | p | p_FDR |
| --- | --- | --- | --- |
| bmi_change | -0.945 | <.001* | <.001* |
| ast_change | -0.186 | .091 | .101 |
| alt_change | -0.378 | <.001* | <.001* |
| isi_bmi_change | 0.663 | <.001* | <.001* |
| Chol Ratio_change | -0.487 | <.001* | <.001* |
| CRP_change | -0.549 | <.001* | <.001* |
| A1C_change | -0.519 | <.001* | <.001* |
| systolic_change | -0.234 | .032* | .038* |
| diastolic_change | -0.094 | .398 | .398 |
| Glucose Fasting_change | -0.614 | <.001* | <.001* |
| Insulin Fasting_change | -0.577 | <.001* | <.001* |
| QUICKI_change | 0.673 | <.001* | <.001* |
| HOMA-IR_change | -0.594 | <.001* | <.001* |
| triglycerides_change | -0.411 | <.001* | <.001* |
| hdl_cholesterol_change | 0.272 | .012* | .016* |
| ldl_cholesterol_change | -0.402 | <.001* | <.001* |
| cholesterol_hdl_ratio_change | -0.468 | <.001* | <.001* |
| IL-10_change | 0.117 | .303 | .319 |
| IL-6_change | -0.414 | <.001* | <.001* |
| adiponectin._change | 0.339 | .002* | .002* |
| Cortical Gray Matter | 0.066 | 0.562 | 0.844 |
| Inferior Parietal | 0.022 | 0.844 | 0.844 |
| Inferior Temporal | 0.023 | 0.842 | 0.844 |
| Lateral Occipital | 0.122 | 0.281 | 0.749 |
| Posterior Cingulate | 0.024 | 0.830 | 0.844 |
| Total Hippocampus | -0.130 | 0.249 | 0.749 |
| AD-Vulnerable Composite | 0.065 | 0.572 | 0.844 |
| Subcortical Gray Volume | 0.264 | 0.018* | 0.143 |

Supplemental Table 6. Metabolic Syndrome in the Weight Loss and Non-Weight Loss Groups.

| **Timepoint** | **WL (n, %)** | **nWL (n, %)** | **OR** | **95% CI** | **p-value** |
| --- | --- | --- | --- | --- | --- |
| Baseline | 37 (68.5%) | 25 (83.3%) | 0.44 | 0.11–1.45 | .196 |
| Follow-up | 12 (22.2%) | 19 (63.3%) | 0.17 | 0.06–0.49 | .0003 |
